# The Mutagenic Impact of Environmental Exposures in Human Cells and Cancer: Imprints Through Time

**DOI:** 10.3389/fgene.2021.760039

**Published:** 2021-10-20

**Authors:** Axel Rosendahl Huber, Arne Van Hoeck, Ruben Van Boxtel

**Affiliations:** ^1^ Princess Máxima Center for Pediatric Oncology, Utrecht, Netherlands; ^2^ Oncode Institute, Utrecht, Netherlands; ^3^ Center for Molecular Medicine, University Medical Centre Utrecht, Utrecht, Netherlands

**Keywords:** mutagens, environmental carcinogens, mutational signatures, cancer, genomics

## Abstract

During life, the DNA of our cells is continuously exposed to external damaging processes. Despite the activity of various repair mechanisms, DNA damage eventually results in the accumulation of mutations in the genomes of our cells. Oncogenic mutations are at the root of carcinogenesis, and carcinogenic agents are often highly mutagenic. Over the past decade, whole genome sequencing data of healthy and tumor tissues have revealed how cells in our body gradually accumulate mutations because of exposure to various mutagenic processes. Dissection of mutation profiles based on the type and context specificities of the altered bases has revealed a variety of signatures that reflect past exposure to environmental mutagens, ranging from chemotherapeutic drugs to genotoxic gut bacteria. In this review, we discuss the latest knowledge on somatic mutation accumulation in human cells, and how environmental mutagenic factors further shape the mutation landscapes of tissues. In addition, not all carcinogenic agents induce mutations, which may point to alternative tumor-promoting mechanisms, such as altered clonal selection dynamics. In short, we provide an overview of how environmental factors induce mutations in the DNA of our healthy cells and how this contributes to carcinogenesis. A better understanding of how environmental mutagens shape the genomes of our cells can help to identify potential preventable causes of cancer.

## Introduction

Somatic mutations accumulate gradually in the DNA of cells as we age (Blokzijl et al., 2016; Cagan et al., 2021). These mutations are incorporated during replication as a result of replicating damaged DNA, error-prone DNA repair or stochastic errors by DNA polymerases (Kunkel and Bebenek, 2000). DNA can be damaged through spontaneous chemical processes, such as hydrolysis causing deamination of nucleotides. In addition, cell intrinsic processes can be mutagenic, such as oxidative stress-induced DNA damage as a consequence of cellular metabolism (Lindahl and Barnes, 2000), stochastic DNA replication errors and expression of APOBEC enzymes that actively deaminate cytosine residues (Roberts et al., 2013), as reviewed in (Tubbs and Nussenzweig, 2017). Besides these endogenous mutagenic processes, exposure to environmental genotoxins can also cause mutagenic DNA damage. The mutagenic action of the different types of DNA damage can be counteracted by the activity of a wide repertoire of DNA repair pathways. Although these repair mechanisms are highly effective, some DNA lesions escape repair, are incorrectly repaired or are fixed as DNA mutations following mispairings generated during replication, resulting in an annual accumulation of 15–40 mutations in healthy human stem cells (Blokzijl et al., 2016). Indeed, loss of DNA repair activity results in a tremendously increased rate of mutation accumulation, depending on the affected pathway and presence of DNA damage (Drost et al., 2017; Zou et al., 2018; Zou et al., 2021; Sanders et al., 2021). Therefore, the mutational landscape of a cell is shaped by a balance between DNA damage induction and the efficiency of the repair thereof (Volkova et al., 2020).

Most of the mutations that accumulate in healthy tissues during normal ageing are induced by endogenous mutagenic processes (Blokzijl et al., 2016; Franco et al., 2018; Lee-Six et al., 2018; Lodato et al., 2018; Osorio et al., 2018; Franco et al., 2019; Lee-Six et al., 2019; Machado et al., 2021). This notion potentially explains why aging is the main risk factor for developing cancer (Edwards et al., 2002; Tomasetti and Vogelstein, 2015). However, in addition to aging, epidemiological data indicates that exposure to carcinogenic environmental exposures greatly increases the risk for developing cancer. For example, 80–90% of lung cancers are thought to be induced by smoking (Peto et al., 2000) and 86% of melanoma cases by UV-radiation (Parkin et al., 2011). Cancer is a global health problem and the leading or second largest cause of premature death in 112 out of 183 countries (Sung et al., 2021). Thus, one of the most effective strategies to prevent cancer is to reduce the exposure of individuals to environmental carcinogens (Emmons and Colditz, 2017). While various carcinogenic environmental agents have been identified, for many cancers the underlying etiology remains unclear. Identification of environmental genotoxins inducing cancer may aid in the design of effective preventive measures (Green et al., 2011; Spira et al., 2017).

Large-scale DNA sequencing of cancer, normal, and cultured cells have revolutionized our understanding of the mutagenic and DNA repair processes that can shape the mutational landscapes in the genomes of human cells. In this review, we will provide an overview of how these DNA sequencing studies have contributed to our understanding on how environmental exposures induce mutations. In addition, we address how the topography of these mutations can provide mechanistic insight into the mutagenicity of environmental genotoxins. Finally, we will discuss how these environmental genotoxins could contribute to the development of cancer, which may be key in the design of strategies to prevent cancer in the future.

## Detection of Mutations Induced by Environmental Genotoxins

Our initial understanding of the mutational consequences of environmental genotoxins relied on several biological assays to assess to mutagenic potential of chemical compounds. Of these, the most well-known is the Ames’ test, which was developed in 1973 (Ames et al., 1975) and is still used for assessing mutagenicity of environmental and medical compounds (Zeiger, 2019). While positivity in the Ames test is a good predictor for mutagenicity as well as carcinogenic potential in rodents (Mortelmans and Zeiger, 2000), bacterial systems do not completely recapitulate the DNA structure and maintenance of mammalian cells (Johnson, 2012). To study mutagenicity in a mammalian context, the use of reporter genes, such as LacZ in mouse models has been employed (Gossen et al., 1989). In this assay, transgenic mice with chromosomally integrated LacZ reporter genes are exposed to mutagens. After the exposure, LacZ fragments are cloned into *Escherichia coli* and inactivating mutations can be selected and quantified as a measure for mutagenicity. However, such an experimental approach is impossible in humans. To overcome this, endogenous reporter genes present in the human genome, such as *HPRT* (Furth et al., 1981) or *TP53* (Pfeifer et al., 2002) have been used. In the *HPRT*-assay, inactivation of the *HPRT* gene by mutations is used as a selection marker. This gene encodes for hypoxanthine-guanine phosphoribosyltransferase (HPRT), which plays a central role in the generation of purine nucleotides through the purine salvage pathway (Sculley et al., 1992). However, cells with HPRT activity also process 6-thioguanine (6-TG) into a toxic guanine analogue, which ultimately leads to cell death (Hayes et al., 2020). The mutagenicity of a compound can be determined by counting the number of cells in an originally HPRT-proficient population that survive 6-TG selection, because they accumulated inactivating *HPRT* mutations (Furth et al., 1981). Further insight into the underlying DNA damaging processes can be obtained by analyzing the spectrum of mutations identified in the reporter genes. In the case of UV-light, induction of specific CC > TT double-base substitutions have been observed in the *HPRT* assay (Hutchinson, 1994)*.* Indeed, sequencing cancer reporter genes, such as *TP53,* revealed the presence of CC > TT mutations in melanoma, suggesting that these mutations were induced by UV-light (Ziegler et al., 1993). In lung cancer, C > A mutations were overrepresented in *TP53* (Hollstein et al., 1991). This difference in mutational spectra between cancer types indicates that different environmental genotoxins caused distinct mutation characteristics. Despite these initial insights, sequencing of reporter genes limits mutation detection to small DNA fragments, which are biased in their sequence makeup and genomic location, making it difficult to extrapolate these findings to the entire genome.

### Sequencing of Cancer Genomes

**FIGURE 1:**
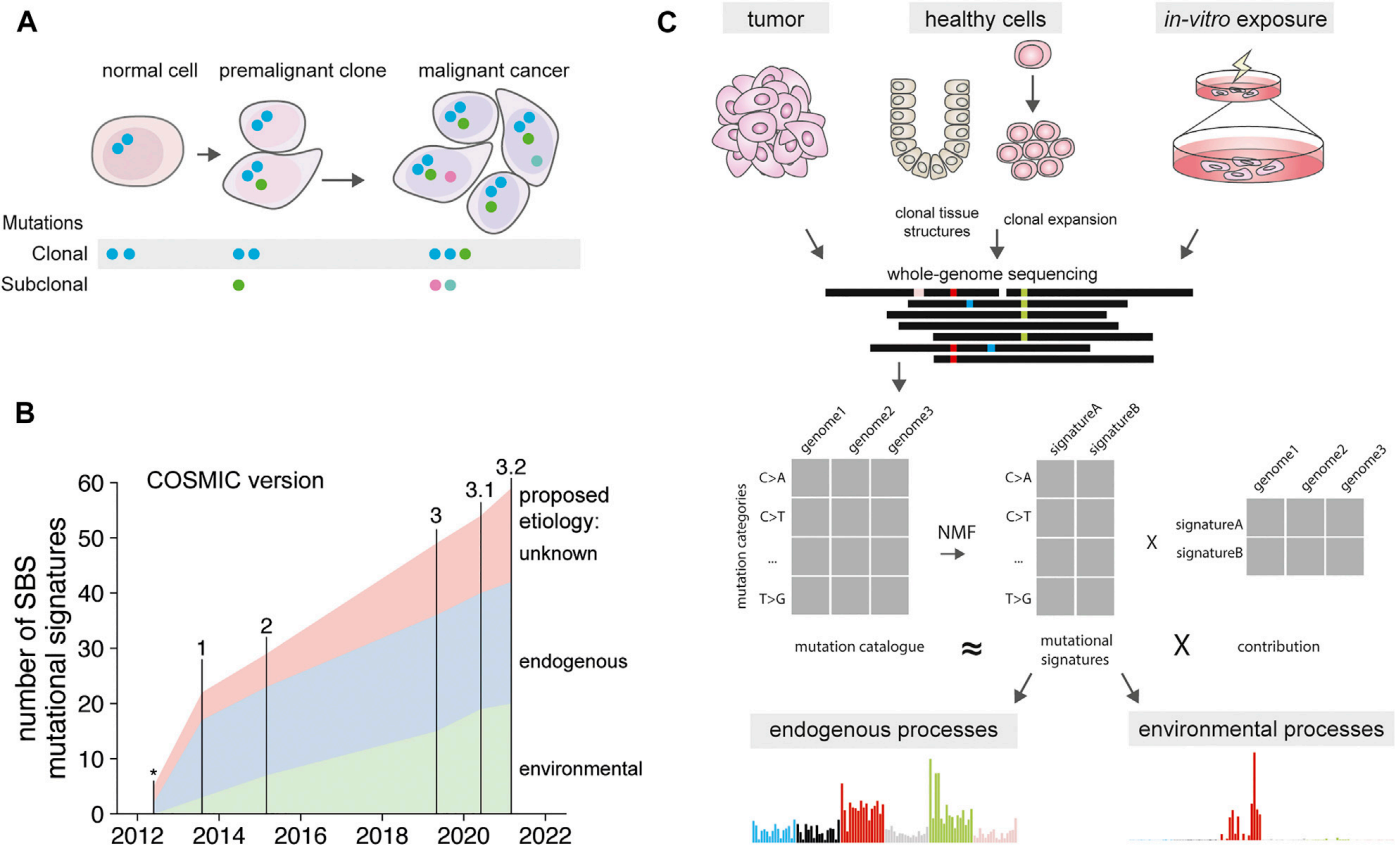
Mutational signatures over time. **(A)** Presence of clonal mutations in normal cells, premalignant clones and cancer. Blue mutations present in the non-malignant normal cell. These mutations are retained in the premalignant clone and cancer, along with additional mutations acquired during tumorigenesis. Subclonal mutations are independently acquired by each cell, and can become clonal when selective sweeps favor expansion of a subclonal cell population. **(B)** Number of discovered mutational signatures catalogued in the COSMIC database, version indicated on top. Signature associations determined as in Supplementary Table S1. *signatures from Nik-Zainal et al., 2012. **(C)** Depiction of mutational signature extraction using tumor cells, normal cells and *in vitro* exposed cells as input source. Signatures can be extracted from the mutation catalogues using non-negative matrix factorization. This results in both signatures, and their relative contribution in each tumor type.

With the advent of next-generation sequencing technologies, mutation detection in whole genomes has become possible. Currently, thousands of cancer exomes and genomes have been sequenced in large consortium-based efforts (Bailey et al., 2018; Priestley et al., 2019b; Campbell et al., 2020). The data of these large-scale genome projects have been made available to the biomedical research community and efforts to increase the number of included patients are ongoing. These collections of somatic mutations are providing an unprecedented amount of information about the activity of mutagenic processes before and after carcinogenesis. As cancer is the result of a clonal expansion originating from a single founder cell, all the mutations present in that ancestral cell will be shared by all cells in the tumor (Nik-Zainal et al., 2012b). Depending on the depth of sequencing and the clonal makeup of the tumor, subclonal mutations from the most predominant subclones can be detected as well (Figure 1A) (Miller et al., 2014; Roth et al., 2014). Thus, mutagenic exposure during the lifetime of the ancestral cell or during early carcinogenesis will be captured in the tumor genome (Nik-Zainal et al., 2012b). Mutagenic processes that are active later after tumor initiation and stochastically present in a single or a small subset of tumor cells are not detectable with traditional cancer genome sequencing methods. Sequencing of single cancer cells is required to accurately detect each subclonal mutation (Wang et al., 2014; Roerink et al., 2018). This approach gives a detailed insight in the mutagenic processes active during tumor progression at individual cell resolution. Clonal expansions of cells also take place *in vivo* during tumor development, such as in the case of tumor relapses, metastases or intratumoral selection pressures (e.g., treatment) favoring the outgrowth of a specific subclone. During these steps, low frequency subclonal mutations in the original tumor can become clonal and detectable by sequencing (Figure 1A) (Priestley et al., 2019a; Li et al., 2019). Both exome and whole genome sequencing can be employed to study cancer genomes. However, whole genome sequencing provides a higher resolution, as it enables the detection of a large number of passenger mutations (Helleday et al., 2014). In addition, whole genome sequencing enables detailed characterization of broad (karyotype), local ploidy changes (loss-off-heterozygosity), and accurate estimates of cancer purity, which is ideally suited for studying mutational spectra and clonal compositions.

### Mutational Signatures

Different mutagenic exposures during the lifetime of a cell can be disentangled by identifying recurrent mutational patterns, or “mutational signatures” across cancer genomes. These mutational signatures are defined by individual mutation classes, such as single base substitutions (SBS), double base substitutions (DBS), or short insertions and deletions (indels). For the most common mutation class, SBS, different mutagenic processes can induce specific base changes, such as UV resulting in C > T mutations and smoking in C > A mutations. These base changes are by convention always reported from the perspective of the pyrimidine base, such as C > A, or T > G, because the strand containing the mutagenic damage cannot directly be deduced. In addition to the type of substitution, the causative processes can display a preference for the direct 5’ and 3’ bases flanking the mutated base. Therefore, single base substitutions are usually depicted as trinucleotide changes, in which the middle base is mutated, resulting in 96 different possibilities (Alexandrov L. B. et al., 2013). When various mutagenic processes are differentially active in a set of tumor genomes, they can be extracted from mutation data using dimension reduction algorithms (Alexandrov L. B. et al., 2013). These algorithms, such as non-negative matrix factorization, reduce the mutation spectra of a multitude of individual cancer genomes into a limited set of recurrent 96-trinucleotide signatures, and the contribution of these signatures in each of the cancer genomes (Figure 1C). The higher the diversity in mutation spectra in the set of assessed genomes, for example, by analyzing across various cancer types with different exposures, and the larger the cohort of samples, the more distinct signatures can be extracted. Indeed, by analyzing increasing numbers of cancer genomes, more and more signatures have been defined in the last decade (Nik-Zainal et al., 2012a; Alexandrov L. L. B. et al., 2013; Alexandrov et al., 2020) (Figure 1B). Each of these signatures is regarded to reflect a specific mutational process (Helleday et al., 2014) and new signatures are still being discovered (Pleguezuelos-Manzano et al., 2020; de Kanter et al., 2021). For many signatures the biological cause remains unknown, for other signatures an underlying molecular association has been proposed and for a subset the underlying causative process has been experimentally confirmed. These molecular causes range from endogenous processes that are active in all cells of the body, to exposure to specific chemotherapeutic agents (Pich et al., 2019; Alexandrov et al., 2020). While the first mutational signatures were composed of single-base substitution patterns (SBS), signatures have now also been defined for double base substitutions (DBS), and indels (ID), and are catalogued as part of the COSMIC database (Alexandrov et al., 2020). Future developments in signature extraction are focusing on the integration of additional genomic characteristics, such as specific genomic regions (Vöhringer et al., 2021), tissue-specific signatures (Degasperi et al., 2020) as well as signatures from structural variants (Li et al., 2020). While most of known signatures have been discovered in cancer genomes, the activity of the underlying mutagenic processes is often not limited to tumor cells alone and can be operative in normal cells.

### Determining Mutation Accumulation in Normal Cells

As a tumor genome can serve as an historical archive, it will contain mutations that were acquired before the onset of tumorigenesis in a precancerous normal cell (Helleday et al., 2014). Most cancers, however, are characterized by a mutator phenotype (Loeb et al., 2003), which may be caused by excessive proliferation, loss of specific DNA-repair components, treatment or distorted cellular metabolism. Therefore, it is difficult to exactly determine which mutations were acquired before the malignant transformation (Stratton et al., 2009). Sequencing the genomes of normal cells can be used to identify which mutagenic processes are already active in normal non-malignant cells. However, detection of somatic mutations in bulk normal tissue is difficult due to the stochastic nature of mutation accumulation and the polyclonal architecture of most tissues. In addition, the amount of DNA of a single cell is not sufficient for standard sequencing technologies and needs to be amplified. To achieve this, three methods have been frequently employed. First, *in vitro* expansion of single stem/progenitor cells into clonal cultures has been used to obtain sufficient DNA of the parental cell (Jager et al., 2018; Rosendahl Huber et al., 2019). This method has been used to determine the mutation accumulation in hematopoietic, intestinal, colorectal, liver, skin, muscle, kidney, and lung cells (Welch et al., 2012; Behjati et al., 2014; Blokzijl et al., 2016; Franco et al., 2018; Lee-Six et al., 2018; Osorio et al., 2018; Franco et al., 2019; Yoshida et al., 2020). While *in vitro* expansion is a highly accurate approach to amplify genomic DNA, as cells are well equipped to copy their own DNA faithfully, and circumvents the need for specialized library preparation methods, it is limited to cells with sufficient replicative potential, such as stem cells. A second approach is based on the microdissection of naturally occurring clonal structures that exist within normal tissues, such as skin, esophagus, colonic crypts, bladder, and endometrium (Martincorena et al., 2015; Martincorena et al., 2018; Brunner et al., 2019; Lee-Six et al., 2019; Yokoyama et al., 2019; Lawson et al., 2020; Moore et al., 2020; Olafsson et al., 2020; Yoshida et al., 2020; Sanders et al., 2021). The downside of this approach is that it relies on the presence of clonal structures or expansions within the tissue of choice, which are not always present, such as in the brain. In addition, only mutations that are present in all cells of the clonal population are captured, whereas more recent mutagenic events are missed. To determine the somatic mutations present in these cells, whole genome amplification (WGA) using strand displacement polymerases has been employed (Lodato et al., 2015; Gawad et al., 2016; Vijg et al., 2017; Lodato et al., 2018). However, WGA-based methods are notorious for introducing amplification biases, resulting in overrepresentation of random loci, and allelic dropouts (Hou et al., 2012). These biases make WGA-based methods less suited for assessing mutations in samples with a relative low mutation burden, such as normal cells. Nonetheless, new promising methods to directly study somatic mutations in single cells have been developed that address amplification-induced artifacts, such as primary template-directed amplification (PTA) (Gonzalez-Pena et al., 2021). PTA relies on the introduction of exonuclease-resistant amplification terminators, resulting in a more uniform amplification of the genome. In addition, duplex-sequencing, such as Nanorate sequencing (Nanoseq), enables the sequencing of two complimentary DNA strands. By comparing the sequences, errors that arise during sequencing can be corrected for, as these are not shared by the two strands. This approach has been employed to detect somatic mutations in cells with no replicative potential, such as differentiated cells (Abascal et al., 2021).

### Mutagenic Processes Active in the Genomes of Normal Cells

Using the methods described above, it has been determined that the genomes of healthy cells gradually accumulate mutations during life (Blokzijl et al., 2016; Lee-Six et al., 2018; Osorio et al., 2018; Machado et al., 2021). This rate varies between ∼40 mutations per year in liver, small intestine, and colon, while hematopoietic stem cells and muscle stem cells are on the lower end with about 13–15 novel mutations each year (Franco et al., 2018; Lee-Six et al., 2018; Osorio et al., 2018). A more pronounced variation has been observed for the mutation spectra and signatures across different organs, suggesting a tissue-specific activity of mutagenic processes. Of these, some processes are active in a clock-like manner in most tissues causing mutation accumulation at a rate that is constant within a tissue (Alexandrov et al., 2015). One of these processes is reflected by signature SBS1, which is induced by spontaneous deamination of methylated cytosines, present in a CpG dinucleotide context, into thymine residues. SBS1 seems more predominant in genomes of fast-dividing cells, such as colon and intestine as well as tumors originating from these tissues (Alexandrov et al., 2015; Blokzijl et al., 2016; Lee-Six et al., 2019). Another clock-like signature, SBS5, is observed in practically all tissues (Alexandrov et al., 2015). Finally, hematopoietic stem and progenitor cells display a unique aging-related signature, termed HSPC signature (Maura et al., 2019; Brandsma et al., 2021). The cause for these latter two signatures remains unknown, but their continuous action in normal cells suggests a role for endogenous mutagenic sources, which are constantly present. In addition to these clock-like processes, the mutational consequences of a variety of environmental mutagenic processes can sometimes be observed in normal cells. For example, in colonic crypts, approximately half of all studied individuals displayed a specific mutational signature, which is characterized by T > N single base substitutions in an ANNT context (of which the underlined base is mutated) and deletions of a single thymidine in short T-homopolymers preceded by adenine (Lee-Six et al., 2019). This signature is caused by exposure to colibactin, a mutagenic toxin produced by *pks + E. coli* (Pleguezuelos-Manzano et al., 2020). These colibactin-induced mutations are shared in phylogenetically related crypts, indicating that these mutations have been induced early during life (Lee-Six et al., 2019). The mutational consequences of *pks + E. coli* can also in rare cases be observed in the genomes of bladder, neuroendocrine and head, and neck cancers. The presence of this mutational signature in these cancers is likely indicative for a history of colibactin exposure, which potentially increased the risk for developing cancer as a direct result of enhanced mutation accumulation (Boot et al., 2020; Pleguezuelos-Manzano et al., 2020). Another example of variable damage induced by environmental exposure is in the skin, where UV-induced mutations can be observed in both melanoma and healthy melanocytes (Tang et al., 2020). In skin not exposed to sunlight, melanocytes harbor a low number of UV-induced mutations. Interestingly, compared to melanocytes present in skin continuously exposed to UV-light, melanocytes from intermittently UV-exposed skin contain a higher number of UV-induced mutations (Tang et al., 2020). This observation suggests that the number of genotoxin-induced mutations does not necessarily has to correlate with the dose of exposure. In addition to melanocytes, the mutational signatures attributed to UV-damage have also been observed in skin-residing lymphocytes (Machado et al., 2021) and sporadically in T-cell lymphoma (Jones et al., 2021), pointing to a past exposure to UV-radiation.

Exposure to genotoxins in cigarette smoke can induce mutations in a patchy manner, affecting some cells but not others, as was recently demonstrated by the sequencing of bronchial epithelial cells in smokers, non-smokers, and ex-smokers (Yoshida et al., 2020). As expected, the non-smokers accumulated aging-related mutations at a constant rate with little variance amongst each assessed cell within the same donor. In contrast, this variance was increased in smokers as well as ex-smokers. Most bronchial epithelial cells displayed a several-fold increase in mutation load, which could be attributed to the tobacco smoke-associated signature SBS4. However, some cells in the lungs of smokers and ex-smokers had no additional mutation load, implying that some bronchial epithelial cells were not or less exposed to the mutagen. Interestingly, these cells with a near-normal mutation load were present in higher numbers in ex-smokers, which may explain why after years of quitting smoking the risk of developing lung cancer is reduced (Yoshida et al., 2020).

### *In Vitro* Assays Mutagenicity

The mutagenic properties of environmental components can also be experimentally determined using cell culture assays. In these experiments, primary cells, cell lines or organoids are exposed *in vitro* to mutagens, followed by clonal expansion and sequencing analysis (Jager et al., 2018). During culture, cells experience both background mutagenesis, which seems to be driven by oxidative stress (Kuijk et al., 2020), and mutation accumulation as a result of the genotoxic exposure. Such *in vitro* experiments can confirm associations between a mutational signature and hypothesized mutagenic exposure (Kucab et al., 2019). Recently, a landmark study has examined the mutational impact of 79 different environmental genotoxins, providing a resource to causally link mutational signatures to specific mutagenic exposures (Kucab et al., 2019). One such carcinogen is aflatoxin B1, which is produced by strains of the fungus *Aspergillus* that grows on contaminated food. This carcinogen is a known mutagen, which induces SBS24 mutations as determined by signature extraction using cancer genomes (Alexandrov L. L. B. et al., 2013) and confirmed by *in vitro* mutagenicity assays (Huang et al., 2017; Kucab et al., 2019). Another carcinogenic compound, aristocholic acid, causes signature SBS22, and has been implicated with bladder and liver cancer (Poon et al., 2015; Ng et al., 2017). Also this association between signature and environmental genotoxin has indeed been confirmed *in vitro* (Nik-Zainal et al., 2015). While aflatoxin B1 and aristocholic acid directly alkylate the DNA, several indirect mutagenic processes have been identified. External exposures can result in unsuccessful apoptosis (Ichim et al., 2015; Hawkins and Miles, 2021) or altered expression of DNA replication and repair enzymes, elevating mutational loads (Russo et al., 2019; Cipponi et al., 2020). As the mutagenicity of these processes has been determined using colony outgrowth assays or reporter gene assays, mutational signatures from these processes are lacking, and could be established in the future using WGS-based methods.

#### Treatment-Induced Signatures

Most chemotherapeutic drugs act by fatally damaging the DNA or blocking the replication thereof (Helleday et al., 2008). However, noncancerous cells can also be damaged by treatment (Quispe-Tintaya et al., 2018). This can result in the accumulation of DNA mutations in normal tissues with potentially adverse effects later in life, such as an increased risk for developing a secondary malignancy (Morton et al., 2019). Indeed, diverse chemotherapeutic drugs have been shown to cause specific mutational signatures. Platinum-based compounds, which cause inter- and intrastrand crosslinks between at guanine-guanine residues, induce mutational signatures SBS31, and SBS35 *in vivo* (Alexandrov et al., 2020) as well as *in vitro* (Boot et al., 2018; Kucab et al., 2019). Other chemotherapies known to induce specific mutational signatures in cancer and *in vitro* are 6-mercaptopurine (6-MP) (Li et al., 2019), which causes SBS87, and 5-fluorouracil (5-FU), which is known to be one of the underlying causes of SBS17 (Christensen et al., 2019; Pich et al., 2019). More recently, it was shown that ganciclovir, a synthetic guanosine analogue used as an antiviral drug to treat reactivation of cytomegalovirus in immunocompromised patients, can induce a highly specific C > A signature at CpA sites in hematopoietic stem and progenitor cells of patients as well as *in vitro* (de Kanter et al., 2021).

The loss of specific DNA repair activity can alter the mutational profile caused by an environmental mutagen. For example, temozolomide exposure has been associated with two different mutational signatures. Patients treated with this alkylating agent can display signature SBS11 in their tumors genomes, which mainly consists of C > T changes in an CpC or CpT context (Alexandrov L. L. B. et al., 2013). However, *in vitro* exposure of induced pluripotent stem cells (iPSCs) yielded a very different signature, which comprised of T > N changes (Kucab et al., 2019). This apparent difference in temozolomide-induced mutational signatures was explained by a defective DNA mismatch repair in the tumor cells of the assessed patients, resulting in SBS11 mutations (Touat et al., 2020).

In contrast, a very similar mutational signature can also be induced by multiple factors, suggesting that some agents induce mutations in an indirect manner. For example, as indicated above, exposure to the chemotherapeutic drug 5-FU/capecitabine can cause SBS17a/b mutations, which are characterized by T > G changes in a CpTpT context (Christensen et al., 2019; Pich et al., 2019). However, this signature is also observed in the genomes of treatment-naïve esophageal and stomach tumors, which was hypothesized to be caused by gastric acid exposure (Dulak et al., 2013; Secrier et al., 2016). In addition, organoids derived from the mouse intestine also accumulate SBS17a/b-like mutations during culturing (Behjati et al., 2014). While the exact underlying mechanism remains unknown, it has been proposed that SBS17a/b mutations might be caused by the incorporation of oxidized guanine residues opposite adenine in the DNA during replication (Tomkova et al., 2018). The similarity between the mutation profiles in (5-FU untreated) tumors exhibiting SBS17a/b mutations and 5-FU exposed organoids suggest that distinct mutation-inducing processes may converge in the same outcome, resulting in similar signatures.

## Topographies of Environmentally Induced Mutations

**FIGURE 2:**
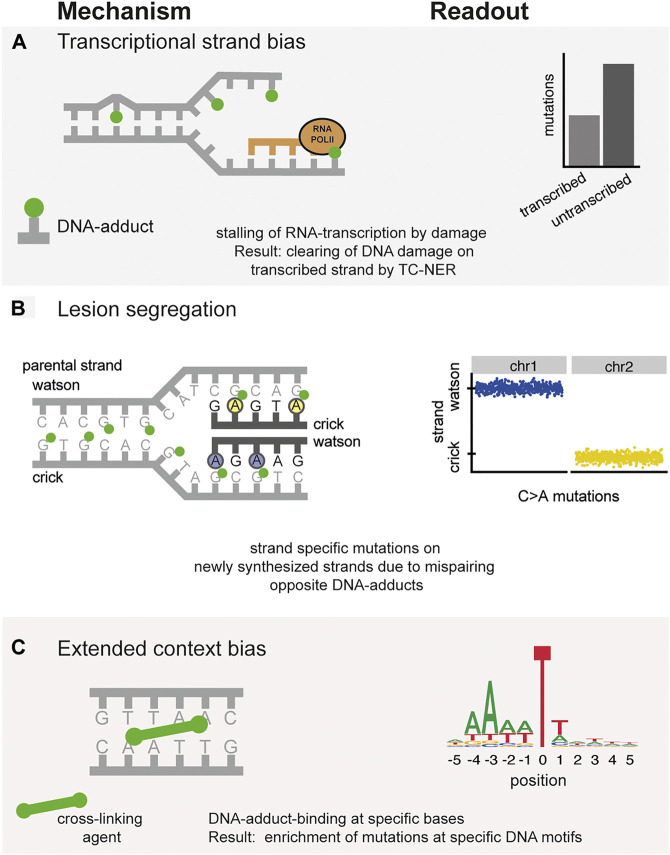
Mechanisms underlying topographical differences in mutation accumulation. Left column: Molecular mechanism of mutation induction. Right column: Typical readout of these processes in mutation data catalogues. **(A)** Transcriptional strand bias. Mutations indicated in green are preferentially repaired by TC-NER on the transcribed strand. **(B)** Incorporation of mutations opposite of DNA-adducts during replication results in the asymmetric division of mutations on either the Watson or Crick strand. **(C)** Preferential binding of DNA-damaging agents to specific contexts, resulting in the extended-context biases of mutations.

*In vitro* experiments can prove causality between exposure to a certain environmental mutagen and a mutational signature observed in the genomes of healthy and/or tumor cells. However, the mechanism by which a mutagen induces mutations cannot always be directly inferred from the signature it causes. Nonetheless, mutations can harbor additional information, which can help in revealing the causative mechanism, such as genomic distribution and strand asymmetries (Figure 2) (Makova and Hardison, 2015; Haradhvala et al., 2016; Aitken et al., 2020). Together, these characteristics form a “mutational topography”, which can yield further insights into the etiology of mutational signatures (Morganella et al., 2016). If the causative factor is known, the topography of a mutational profile can help to understand the molecular mechanism by which the factor induced mutations. In the following paragraphs, we will highlight some of the most informative features important for the study of environmentally induced mutations.

### Transcriptional Strand Asymmetry

One of the major conformation-changing processes that occurs in DNA is its transcription into RNA. This process requires a temporary separation of the two DNA strands to synthesize a complementary RNA molecule. When RNA polymerase II (RNA Pol II) encounters a blocking DNA lesion, it cannot proceed and will stall (Vermeulen and Fousteri, 2013). To continue transcription, RNA Pol II recruits transcription-coupled nucleotide excision repair (TC-NER), which initiates the repair of the blocking lesion. As this process is dependent on transcription, TC-NER can only take place in actively transcribed regions of the genome. Importantly, only the transcribed (template) strand will repaired be by TC-NER. This preferential repair of the transcribed strand results in a characteristic mutational strand asymmetry in expressed regions (Green et al., 2003; Haradhvala et al., 2016) (Figure 2A). The bias of the mutational strand asymmetry (i.e., which base is on the transcribed strand and thus protected) can indicate which nucleotide of the mutated base pair was originally damaged. For instance, the mutational signature induced by smoking, SBS4, displays a depletion of C > A mutations when the guanine of that mutated base pair was on the transcribed strand. This bias indicates that damaged guanine residues underlie the signature as these are preferentially repaired on the transcribed strand. Indeed, mutagenic agents in tobacco smoke, such as benzo(a)pyrene, are known to react with guanine and thereby damaging the DNA. Thus, the presence of a transcriptional strand bias in a specific signature can give clues into the DNA damage that cause specific signatures.

### Influence of DNA-Replication State

Recent studies implicate that the replicative state of a cell is an important determinant for incorporating chemotherapy-induced DNA damage (Pich et al., 2021). According to this model, the turnover of damaged nucleotides is so quickly, that only cells in a replicative state during chemotherapy exposure will incorporate damaged nucleotides during DNA-replication. After DNA replication, damaged nucleotides are converted in mutations. This mechanism might be important in more mutagenic exposures where the damaged nucleotides are concerned, such as in thiopurine treatment, which also can induce a mutational signature (Li et al., 2019). In the future it might be feasible to take this mechanism into account during chemotherapy treatment to spare healthy replicating cells.

### Strand Lesion Segregation

When DNA-lesions encounter a replication fork, these lesions can cause mispairing (Boiteux and Jinks-Robertson, 2013). The mutagenicity of such lesions can depend on the activity of different polymerases on the leading and lagging strands (Seplyarskiy et al., 2018). DNA replication can result in asymmetric distribution of mutations between Watson and Crick strands after short mutagenic exposures. During DNA replication, mispairing opposite damaged bases on the parental DNA strand can result in mutations in the newly synthesized strand of DNA in a strand-specific manner (Figure 2B). This strand lesion segregation was first observed in mice receiving the highly mutagenic agent diethylnitrosamine (DEN), which induces liver cancer (Aitken et al., 2020). As the exposure to DEN was a single short burst, a clear separation of mutations in a Watson-Crick strand asymmetry could be observed in the genomes of the liver tumors. It is supposed that such a mechanism is involved during every DNA replication when damaged bases result in mispairing during DNA synthesis. However, a clear strand-specific separation is generally not visible in the genomes of tumor and normal cells, as exposure to a mutagenic source across multiple cell divisions will result in mutation accumulation on alternating Watson and Crick strands (Aitken et al., 2020). This prolonged exposure will result in DNA damage on both Watson and Crick strands, and therefore in most cancer genomes this mutational asymmetry is not observed. However, strand lesion segregation is observed in renal, liver and biliary tract-tumors containing aristocholic acid or aflatoxin exposure (Aitken et al., 2020). This segregation therefore indicates that the exposure to these environmental genotoxins was limited to a single replication round.

### Double Strand Breaks Induced by Radiation

Ionizing radiation particles have the potential to induce DNA double strand breaks (DSBs) in the genomes of affected cells. These DSBs, when repaired incorrectly, can result in short insertions or deletions, and larger chromosomal aberrations. Indeed, in radiation exposed cells, mice and tumors indel mutations and larger structural variants displaying the hallmarks of erroneous DSB repair can become visible (Behjati et al., 2016; Rose Li et al., 2020; Kocakavuk et al., 2021). While there is no single hallmark of radiation-induced cancers, and mutation induction may vary on the specific type of radiation (Rose Li et al., 2020), detection of radiation-induced mutations post-treatment could be used to determine sensitivity to radiation in the future (Kocakavuk et al., 2021).

### Variation in Regional Mutation Burden

Different chromatin states, gene expression, timing of DNA replication during the S-phase and differential DNA repair across the genome can result in regional differences in the density and types of mutations (Makova and Hardison, 2015; Supek and Lehner, 2015). These regional mutation densities are so specific that the distribution of mutations across cancer genomes can be used to predict the tissue type of origin of tumors (Jiao et al., 2020). In addition, the three-dimensional conformation of the genome influences regional mutation rates at boundaries between different genomic topologically associating domains (Akdemir et al., 2020). In addition, steric hindrance of DNA-binding molecules can impair the repair of DNA damage at specific DNA loci. In melanomas, it has become apparent that transcription factor-binding sites are enriched for mutations, as nucleotide excision repair is not able to access these sites resulting in mutations enriched at transcription-factor binding motifs (Perera et al., 2016; Sabarinathan et al., 2016).

### Extended Mutation Context

Some mutagenic processes display a clear preference for a specific context beyond the direct 5’ and 3’ flanking bases. Assessing an additional base on each side of the mutation results in a 1,536 different pentanucleotide categories. While it is technically possible to delineate signatures using pentanucleotide changes, spreading mutations across a much larger number of categories results in much sparser data, complicating analyses (Alexandrov L. B. et al., 2013). Moreover, nucleotides even further away from the mutated base may be specifically enriched at mutated bases, suggesting a mechanistic cause. Such an extended context has been demonstrated for mutations induced by the cytidine deaminase enzyme APOBEC. These mutations are enriched at DNA hairpin sites, indicating that these secondary structures provide an optimal substrate for APOBEC enzymes (Buisson et al., 2019; Langenbucher et al., 2021). In addition, the mutations induced by colibactin-producing *pks* ^*+*^ *E. coli,* which are mainly T > N SBS mutations display a striking enrichment for adenines at the -3 position. As colibactin is known to preferentially bind to adenine (Wernke et al., 2020), the broader sequence context suggests that colibactin causes a cross-link between the -3 positioned adenine and the adenine opposite the mutated base (Boot et al., 2020; Pleguezuelos-Manzano et al., 2020) (Figure 2C).

## Cancer Driver Induction by Environmental Genotoxins

### Induction of Driver Gene Mutations

Environmental genotoxins have the potential to induce many somatic mutations, often recognizable by the mutational signatures they leave behind in the genomes of cells (Kucab et al., 2019). However, the presence of such a mutational signature does not necessarily mean that the exposure contributed to carcinogenesis. As mutations in specific driver genes are required for carcinogenesis, evidence of driver gene induction by environmental mutagens could be used to obtain additional evidence to link environmental genotoxins to the initiation of cancer. The specific type and context characteristics of driver mutations can be used to attribute cancer driver mutations to specific mutational signatures (Poulos et al., 2018; Temko et al., 2018). In addition, the topography of mutations can provide further evidence for the involvement of specific genotoxins in causing the oncogenic mutations driving cancer. In the colorectal cancer-driving *APC* gene, 5.3% of the mutations display the extended motif characteristic for colibactin-induced mutations (SBS88/ID18), implying a causative role for *pks* ^*+*^ *E. coli* in the induction of colorectal cancer (Pleguezuelos-Manzano et al., 2020; Terlouw et al., 2020). However, the analysis of all *APC* mutations in a large colorectal cancer cohort indicates that most mutations are C > T substitutions at CpG sites, pointing to a major role for the endogenous clock-like deamination of methylated cytosines (SBS1) in the induction of cancer driving mutations (Blokzijl et al., 2016). However, even if a relatively large fraction of driver mutations is induced by endogenous processes, a tumor harbors typically between 2 and 10 cancer driver mutations (Martincorena et al., 2017). Additional driver mutations can be induced by environmental genotoxins on top of the driver mutations caused by endogenous processes, which may be sufficient to induce full malignancy. In this scenario, environmental carcinogens may be responsible for the induction of a larger fraction of cancers than suggested by the fraction of driver mutations linked to these carcinogens (Tomasetti et al., 2017; Volkova et al., 2020).

### Driver Mutations in Non-malignant Tissue

Despite the well-established role of driver mutations in cancer, recent sequencing studies have shown that normal tissues can harbor many cancer driver mutations without being malignant. In 83% of skin naevi (common moles), oncogenic *BRAF* V600E mutations have been found (Pollock et al., 2003). While melanoma can originate from a naevus, most naevi never progress to melanoma. Moreover, in the skin of eyelids of elderly individuals between 55 and 73 years of age, 18–32% of small clonal populations of skin cells can contain mutations in classical cancer driver genes, such as *NOTCH1* and *TP53*. These mutations might underlie the clonal expansion of these driver-containing cells, but the tissue is phenotypically normal. Deep-sequencing of esophageal epithelium revealed a similar presence of clonal expansions, which contained cancer driver mutations in the same genes as found in normal skin (Martincorena et al., 2015; Martincorena et al., 2018; Yokoyama et al., 2019). While some of these expansions can be detected in young individuals, their frequency and size are increased in older individuals. Despite the presence of these driver mutations in up to 30% of the cells, the tissue still functions normally and appears nonmalignant. A similar clonal expansion of cells has been observed in the hematopoietic system, named clonal hematopoiesis (CH). The incidence of CH is higher in elderly individuals and was initially detected by somatic mutations in *DNMT3A*, *ASXL1,* and *TET2* (Genovese et al., 2014; Jaiswal et al., 2014). These genes are frequently mutated in leukemia and are considered to have a leukemic driving potential (Martínez-Jiménez et al., 2020). Later studies discovered CH without driver mutations is highly prevalent (Zink et al., 2017), raising the question whether CH is a pre-cancerous state. These observations raise the question whether these clonal expansions represent pre-cancer states, or if the enhanced clonality of a tissue is a characteristic of normal aging (Brash, 2015; Colom et al., 2021).

## Discussion

Sequencing the DNA of tumor, healthy and *in vitro* exposed tissues has provided a wealth of insights into the mutagenicity of environmental genotoxins, and the mechanisms by which they might contribute to carcinogenesis. These insights can be particularly useful to identify factors that increase the risk for developing cancer and help to design preventive measures. During the last decade, tremendous gains in knowledge have been achieved by whole genome analysis of somatic mutations. Signatures can be used to identify past mutagenic processes using somatic mutation data alone. Hypothesized causes of these signatures can be experimentally tested using experimental setups (Kucab et al., 2019). In comparison to many endogenous signatures, which can provide information on the presence of genetic predisposition (Drost et al., 2017), and acquired targetable vulnerabilities (Davies et al., 2017; Nguyen et al., 2020), environmental signatures are not (yet) used in clinical decision-making (Van Hoeck et al., 2019). However, presence of certain environmental signatures can be associated with clinical outcomes, as in the case of radiotherapy-induced deletions in metastatic cancer, which are associated with a poor prognosis (Kocakavuk et al., 2021).

As the genomics revolution has greatly increased our knowledge on mutation accumulation in our cells, it might be reasoned that now most major mutational processes are known. However, new signatures are still being discovered by analyzing genomes of healthy cells as well as cancer (Pleguezuelos-Manzano et al., 2020; de Kanter et al., 2021; Gurjao et al., 2021). An example of using somatic mutation data to assess cancer risk in exposed humans*,* is the recent study on Chernobyl survivors (Morton et al., 2021; Yeager et al., 2021). In these studies, the mutagenic effects of ionizing radiation could be observed in papillary thyroid carcinoma, but no transgenerational germline *de novo* mutations could be detected. Monitoring of individuals who have been exposed to high doses of environmental genotoxins, such as cancer survivors, may help to assess risk of developing cancer. In addition to targeted assessment of potential risk groups, large scale cancer genomic datasets should focus on obtaining the most diverse dataset possible, as such a dataset will capture a wider variety of cancer-causing exposures (Balmain, 2020; Ginsburg et al., 2021).

**FIGURE 3:**
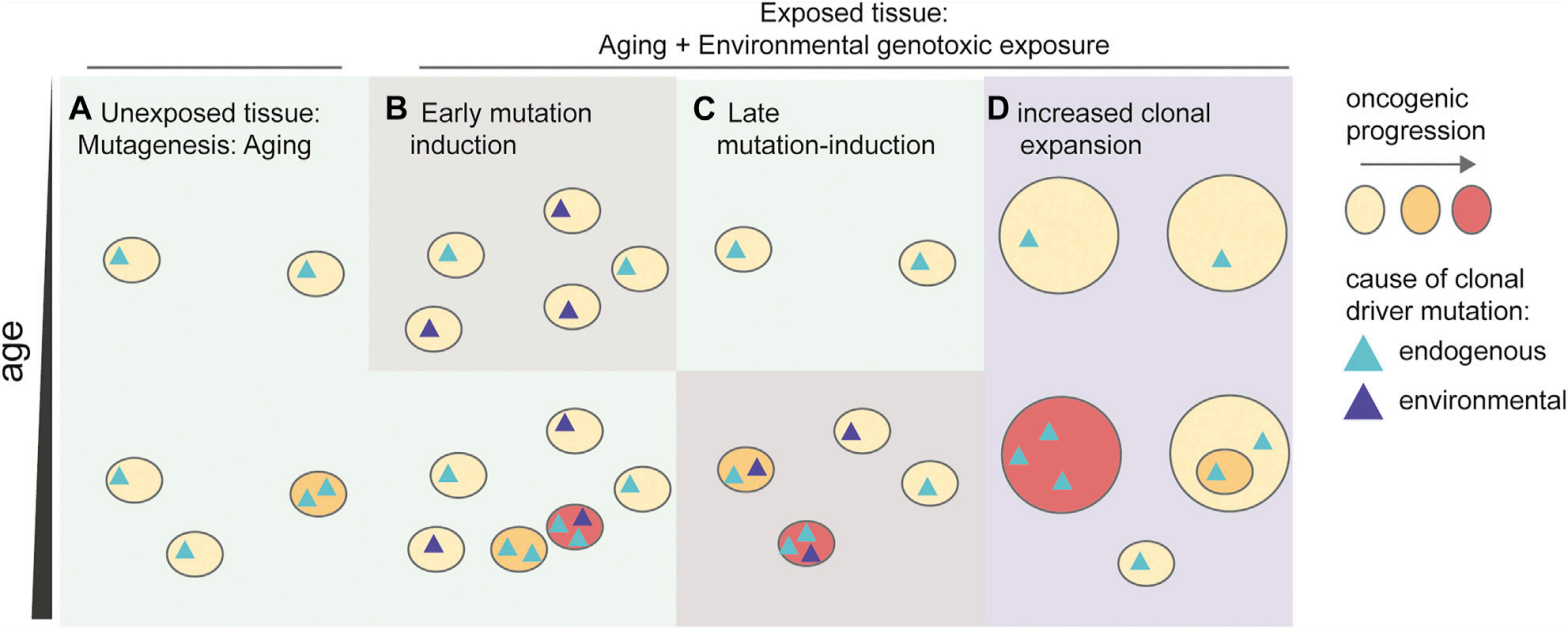
Influence of environmental genotoxins in the development of cancer. Schematic depicting multiple possible mechanisms responsible for an increased risk on developing cancer. **(A)** Aging drives a general mutation accumulation, inducing early driver mutations, which drive clonal expansions. These age-related clonal expansions induce an increasing, but relatively low risk on developing cancer during aging. **(B)** Early mutagenesis induces additional clones containing driver mutations in the tissue. An increased number of driver-containing clonal expansions increases the chance of acquiring additional driver mutations resulting in malignant cancer (red). **(C)** Late mutagenesis by environmental factors induces additional mutations in pre-existing oncogenic clones resulting in malignancies (red). **(D)** Exposure to the environmental factor results in preferential selection of driver-containing clones. The enhanced size of the clones increases the chance of acquiring additional driver mutations, resulting in malignancy.

As discussed above, clonal expansions with and without driver mutations are highly prevalent in multiple tissues, and the incidence of these clonal populations increases as we age (Figure 3A). While some clonal expansions harbor driver mutations, these expansions are not malignant, as they lack the hallmark characteristics of cancer (Hanahan and Weinberg, 2011). Here, we propose three mechanisms how environmental genotoxins may accelerate the onset of cancer (Figure 3). First, exposure to environmental mutagens early in life can result in expansion of a subset of mutated cells. Later during life these cells may expand further and develop into cancer (Figure 3B). Enhanced mutagenesis can also play a role in later stages of cancer development, as environmental genotoxins can induce additional driver mutations in cells, or cell populations already containing a single or low number of driver mutations, resulting in cancer (Figure 3C). Finally, other rate-limiting steps beyond mutation induction can be involved. As stated above, clonal expansions are highly prevalent in the population, and may precede cancer onset. Thus, exposure to environmental genotoxins can alter cellular selection and promote the expansion of clones that contain specific driver mutations (Figure 3D). This mechanism does not require a direct induction of driver mutations by the genotoxin. Indeed, it was recently shown that a large number of carcinogens does not cause an elevated mutation load or specific mutational signatures (Riva et al., 2020), raising the question how cancer is induced, if not *via* mutation induction.

A potential mechanistic explanation for non-mutagenic cancer induction has been proposed in two studies on therapy-associated AML (t-AML), which pointed to a causal role for chemotherapy *via* selection of cells that are resistant to cytotoxic DNA damage. Two genes involved in the induction of apoptosis after DNA damage, *TP53,* and *PPM1D* were more frequently mutated in t-AML compared to *de novo*, treatment-naïve AML (Wong et al., 2015; Hsu et al., 2018). The enrichment of *TP53* and *PPM1D* mutations in t-AML can be explained by preferential selection of pre-existing HSCs, which already harbor *TP53* and *PPM1D* mutations prior to chemotherapy exposure. As both genes are involved in inducing apoptosis because of elevated DNA damage levels, cells that lack the function of these genes may have an increased chance to survive the genotoxic stress induced by cancer treatment. Indeed, pre-existing clones containing *TP53* mutations can be detected in patients before treatment (Wong et al., 2015) and genotoxic exposure, such as radio- and chemotherapy, can alter selection dynamics favoring survival and expansion of these mutated cells (Bolton et al., 2020). This non-mutagenic promotion of clonal populations containing driver mutations may be an explanation why some carcinogens may induce cancer without inducing any additional mutations (Riva et al., 2020).

In humans, the incidence of cancer is low during reproductive age, presumably due to evolutionary pressure (de Magalhães, 2013). From an evolutionary perspective, however, there is no added benefit for reducing pre-malignant states cells beyond the threshold of malignant cancer induction. Therefore, there is no requirement to inhibit clonal expansions containing driver mutations if the tissue still functions normally (Martincorena and Campbell, 2015; Martincorena et al., 2018). However, aging, or exposure to environmental mutagens can both lead to elevated mutation levels and/or alter clonal dynamics. These additional processes may be enough to accumulate the additional characteristics and reach the “tipping point”, where cells become malignant (Lee-Six, 2018). To reach this tipping point, a relatively modest increase in mutation load, or promotion of pre-cancerous expansions by environmental factors may already be enough to increase the incidence of cancer (Tomasetti et al., 2017; Volkova et al., 2020). Now is the time to systematically assess which environmental genotoxins can induce cancer, and in which manner. Determining how these agents induce mutational signatures or alter clonal tissue dynamics could lead to further insights, and aid in the design of future strategies in the prevention of cancer.

## Supplementary Material

Supplementary Table S1 Mutational signatures and their suggested etiologies over time.
